# Pica in Childhood: Concurrent and Sequential Psychiatric Comorbidity

**DOI:** 10.1002/eat.24491

**Published:** 2025-06-27

**Authors:** Laura G. Rubino, Cynthia M. Bulik, Samuel J. R. A. Chawner, Nadia Micali

**Affiliations:** ^1^ Department of Psychological and Brain Sciences Drexel University Philadelphia Pennsylvania USA; ^2^ Centre for Neuropsychiatric Genetics & Genomics, School of Medicine Cardiff University Cardiff UK; ^3^ Department of Psychiatry University of North Carolina at Chapel Hill Chapel Hill North Carolina USA; ^4^ Department of Medical Epidemiology and Biostatistcs Karolinska Institutet Stockholm Sweden; ^5^ Department of Nutrition University of North Carolina at Chapel Hill Chapel Hill North Carolina USA; ^6^ Center for Eating and Feeding Disorders Research Mental Health Services of the Capital Region of Denmark, Psychiatric Centre Ballerup Copenhagen Denmark; ^7^ Institute of Biological Psychiatry Psykiatrisk Center Sct. Hans Roskilde Denmark; ^8^ Great Ormond Street Institute of Child Health University College London London UK

**Keywords:** ADHD, ALSPAC, comorbidity, DAWBA, eating disorders, pica

## Abstract

**Objective:**

Pica is the persistent eating of nonnutritive, nonfood substances, and is associated with serious medical consequences. There has been a lack of research into the psychiatric comorbidities of pica, despite being important for informing clinical care. The current study examines psychiatric comorbidities of pica in childhood and the longitudinal relationship between childhood pica and adolescent eating disorders.

**Method:**

We analyzed data from the Avon Longitudinal Study of Parents and Children study. Pica and psychopathology, assessed with the Development and Well‐Being Assessment and the Strengths and Difficulties Questionnaire, were assessed at about 7‐ and 10‐years of age, and reported eating disorders (EDs) at 14‐, 16‐, and 18‐years of age. We conducted linear and logistic regression models, adjusting for covariates, to identify concurrent psychiatric comorbidities, as well as risk for later EDs. We conducted the Benjamini–Hochberg correction procedure to correct for multiple testing.

**Results:**

Pica (prevalence ranged from 0.33% to 2.33% dependent on age) was associated with increased odds of any psychiatric disorder and behavioral disorders in early childhood (OR = 7.30, *q* < 0.001, and OR = 5.65, *q* < 0.001, respectively) and mid‐childhood (OR = 5.75, *q* < 0.001, and OR = 10.66, *q* < 0.001, respectively), and greater concurrent hyperactivity, conduct problems, peer problems, prosocial difficulties, and emotional difficulties (*q* < 0.01 across analyses). We did not find evidence that pica presence increased odds for concurrent emotional disorders nor for later ED risk.

**Discussion:**

The association between pica and psychiatric and behavioral disorders indicates a likely shared etiology. Our findings provide insight into the psychiatric characteristics of children with pica and highlight they may require complex behavioral support beyond their eating difficulties.

**Summary**

- This study investigated the relationship between pica (the eating of nonfood substances) and mental health conditions in childhood.
- We found that in childhood, pica was associated with increased odds of having behavioral disorders (e.g., attention deficit/hyperactivity disorder [ADHD] and conduct disorder) and any psychiatric disorder (e.g., anxiety or ADHD) but not emotional disorders (e.g., obsessive‐compulsive disorder [OCD] and depression).
- Our findings suggest children with pica may require complex behavioral support beyond their eating difficulties.

**Abbreviations**
ADHD: attention deficit hyperactivity disorder
ALSPAC: Avon Longitudinal Study of Parents and Children
AN: anorexia nervosa
BED: binge eating disorder
BN: bulimia nervosa
DAWBA: Diagnostic and Well‐Being Assessment
DSM‐5: Diagnostic and Statistical Manual of Mental Disorders‐Fifth Edition
ED: eating disorder
PD: purging disorder

## 1 Background

Pica, the eating of nonfood substances, occurs across human populations and life stages. Sometimes pica behaviors are considered beneficial. For example, geophagy is common in some cultures among pregnant women and it is thought to have nutritional benefits (Young and Miller 2019). However, sometimes pica behaviors can have clinical, and potentially negative, impacts, in which case, a diagnosis of pica would be warranted. According to the Diagnostic and Statistical Manual of Mental Disorders (DSM‐5), pica is characterized by the “persistent eating of non‐nutritive, nonfood substances over a period of at least one month” (American Psychiatric Association [APA] 2013).” To warrant a diagnosis, the eating behavior must be inappropriate relative to the developmental level of the individual and not part of a culturally accepted practice. Further, if the eating behavior co‐occurs with another psychiatric or medical condition or pregnancy it must be severe enough to garner additional clinical concern (DSM‐5; APA 2013). Pica is associated with serious medical side‐effects, such as ruptures or obstructions in gastrointestinal systems, anemia, and increased mortality (Decker 1993; Dumaguing et al. 2003; McLoughlin 1988; Syrakos et al. 2008). From here within, when we use pica we refer to behaviors warranting a DSM‐5 diagnosis, when the behavior is outside cultural norms and is potentially harmful. Despite potentially serious consequences, pica remains understudied. It is essential we increase our understanding of pica and who might be more at risk of engaging in pica to inform identification and clinical management.

Studies investigating the prevalence of pica in children are heterogeneous regarding setting, sample characteristics, and diagnostic criteria, and report a range of prevalence estimates from 1.7% to 5.0% in typically developing child populations (Hartmann et al. 2018; Marchi and Cohen 1990; Murray et al. 2018) and indicate pica prevalence might ameliorate as children grow older (Papini et al. 2024). Pica has been found to be more common in autistic children than non‐autistic children throughout development (Emond et al. 2010; Papini et al. 2024). For example, one study found pica behaviors were present in 23% of autistic children but 3.5% in population‐based controls (Fields et al. 2021), and another found pica present in 10%–13.6% of children with autism but 0.25%–2.22% of children without autism (Papini et al. 2024). Among those with developmental disabilities, prevalence ranged from 0.98% to 3.5%, whereas among those without developmental disabilities, prevalence ranged from 0.21% to 2.08% (Papini et al. 2024). However, it should be emphasized that pica does not exclusively occur in children with neurodevelopmental conditions and is present in neurotypical children (Fields et al. 2021; Papini et al. 2024). The varying prevalence estimates may reflect true population differences and/or differences across developmental stages, but could also reflect methodological differences. To increase our understanding of who engages in or might be at risk for pica behavior, we need to better characterize the relationship between pica and other childhood psychopathology both concurrently and longitudinally (across development).

Although behaviors associated with pica have been linked to various behavioral and emotional disorders through case studies and hypothesized theoretical models, there is a lack of empirical large‐scale studies investigating the association between pica and psychopathology. For example, pica has been conceptualized as a potential compulsive behavior (Bhatia and Gupta 2009; Stein et al. 1996). Case studies found treatment for obsessive compulsive disorder (OCD) led to a reduction in pica behavior (Bhatia and Gupta 2009; Hergüner et al. 2008), but to our knowledge no study has investigated the prevalence of pica among children with OCD, nor whether children with pica are more likely to have OCD. Additionally, pica has also been conceptualized as being a disorder of impulse control (Saddichha et al. 2012). Case studies report medication treatment for attention‐deficit/hyperactivity disorder (ADHD) decreased pica behavior (Hergüner and Hergüner 2010; Saddichha et al. 2012; Vieira and Castello Branco 2023). However, in both autistic children (Neumeyer et al. 2019) and neurotypical children (Hartmann et al. 2018), pica was not significantly correlated to ADHD or hyperactivity, respectively. Furthermore, we are unaware of any studies directly investigating the relationship between pica and anxiety or depression in children.

There is mixed evidence that pica is cross‐sectionally associated with other eating disorders (EDs) and eating disorder behaviors. Although one study reported pica was not associated with dietary restraint and weight and shape concerns in children (Hartmann et al. 2018), other studies found pica was associated with greater fears of weight gain, dietary restriction, dissatisfaction with shape and weight, purging, and binge eating in children (Murray et al. 2018) and adults (Hartmann et al. 2022). In the context of anorexia nervosa (AN), some individuals eat ice and nonfood items, but whether pica precedes or antecedes the development of AN is unclear (DSM‐5). Work is needed to confirm the relationship between pica and whether pica behavior in childhood is associated with later ED diagnoses (i.e., sequential comorbidity).

The current study aims to investigate the overlap between pica and other psychiatric conditions. The first aim of the study is to examine the relationship between the presence of pica in early and late childhood and the presence of other psychiatric diagnoses in early and late childhood (e.g., ADHD, OCD, anxiety). The second aim is to examine the prospective association between pica in childhood and eating disorder diagnoses in mid to late adolescence.

## 2 Method

### 2.1 Participants

We accessed data from the Avon Longitudinal Study of Parents and Children (ALSPAC), a cohort‐based longitudinal study that invited all pregnant women expected to give birth during April 1, 1991, and December 31, 1992, living in or near Avon, UK, to participate. 14,541 children from these pregnancies were enrolled; 13,988 children were alive at 1 year. At the 7‐year assessment, 713 more children were enrolled. At the late childhood (after 7‐year) assessment, 151 additional children were enrolled, for a total of 15,447 pregnancies included after age 7. Data were available from 13,906 children at the 7‐year assessment, 12,772 children in late childhood (> 7 and < 13 years old), 12,254 during adolescence (13–16 years old), and 11,875 children from the transition to adulthood period (16–18 years old; Boyd et al. 2013; Fraser et al. 2013). The total sample size for analyses using any data collected after the age of seven is therefore 15,447 pregnancies, resulting in 15,658 fetuses. Of these, 14,901 children were alive at 1 year of age. All women gave informed consent. Informed consent for the use of data collected via questionnaires and clinics was obtained from participants following the recommendations of the ALSPAC Ethics and Law Committee at the time. Teenagers were reconsented when they reached 18 years of age. If consent was withdrawn at any time point, the participant's data were not used from that time point on. Ethical approval for the study was obtained from the ALSPAC Ethics and Law Committee and the Local Research Ethics Committees. For this study, we excluded children for which sex, that is, sex assigned at birth, data were not available. Further, we removed one twin from twin pairs at random (Micali et al. 2015).

### 2.2 Procedure

Mothers were recruited through media advertisements and conversations at community locations and with doctors at maternity health services. Once enrolled and consented, there were frequent assessments between the child's birth and their turning 18 years of age. Assessments included child‐completed questionnaires, clinical assessments, and assessments about the child completed by the mother or other main caregiver (Boyd et al. 2013). The current study uses questionnaires the mother or main caregiver completed about pica behaviors in their child at the 36‐, 54‐, 65‐, 77‐, and 115‐month assessments and psychiatric disorders in their child at the 7‐year (81 and 91 months) and 10‐year (115 and 128 months) assessments; eating disorder diagnoses were obtained from child, parent, and objective data at the 14‐, 16‐, and 18‐ year wave (Micali et al. 2015). Please note that the study website contains details of all the data that are available through a fully searchable data dictionary and variable search tool: http://www.bristol.ac.uk/alspac/researchers/our‐data/. Please see Supporting Information Table 1, which outlines the timing of the current study measures and see https://www.bristol.ac.uk/alspac/researchers/our‐data/timeline/ for a timeline of ALSPAC.

### 2.3 Measures

#### 2.3.1 Pica

At 36‐, 54‐, 65‐, 77‐, and 115‐month mothers were asked, “How often does the child eat coal, dirt, or other nonnutritious substances?” Responses options were rated on a scale of one to four, representing “yes, every day,” “yes, at least once a week,” “yes, less than once a week,” and “no, not at all,” respectively. If respondents reported at least “yes, less than once a week,” they were asked a follow‐up question to clarify what the child ate to ensure understanding of the question. Following previous work, we created a binary pica yes/no value at each time point (see Papini et al. 2024 for details). We also created a variable indicating persistent pica (pica_pers:_ if pica was present at two timepoints or more across the five assessments) and any pica (pica_any:_ if pica was present at least at one timepoint).

#### 2.3.2 Psychiatric Disorders

At the 91‐ and 128‐months (child's age; about 7.50 and 10.67 years, respectively) assessments, parents completed the Development and Well‐Being Assessment (DAWBA; Goodman et al. 2000) answering questions about their child, to generate International Classification of Diseases‐10 (ICD‐10) and Diagnostic and Statistical Manual of Mental Illness, fourth edition (DSM‐IV) psychiatric diagnoses. For this study, the DAWBA was adapted to be a questionnaire instead of a structured interview designed to generate the likelihood of the presence of psychiatric illnesses (University of Bristol). Following an established and validated approach, a computerized algorithm was applied to the caregiver responses, deriving the likelihood of a diagnosis, scored on a six‐point scale based (0 = 0.1%; 1 = ~0.5%; 2 = ~3%, 3 = 15%, 4 = 50%, 5 = ≥ 70%; Goodman et al. 2000). From these likelihoods, a binary yes/no presence of disorder was derived.

#### 2.3.3 Dimensional Psychopathology

At the 81‐ and 115‐month assessments of child age (about 6.75 and 9.78 years of age, respectively), mothers or main caregivers completed the strengths and difficulties questionnaire (SDQ; Goodman 1997). The total scores for the emotional symptoms, conduct problems, hyperactivity/inattention, and peer relationship problems subscales range from 0 to 10, with higher scores indicating greater psychological difficulties. The prosocial behavior subscale is also scored on a scale of 0–10, but higher scores indicate lower psychological difficulties. A total difficulties score consisting of all subscales but the prosocial scale was also computed; scores range from 0 to 40, with higher scores indicating greater difficulties.

#### 2.3.4 Eating Disorder Symptoms

At the 14‐, 16‐, and 18‐year marks, youth completed adapted versions of the Youth Risk Behavior Surveillance System questionnaire that assessed for ED behaviors and objective body mass index (BMI) during in‐person assessments. The questionnaire assessed binge eating with two questions examining the frequency of eating a large amount of food in a short period of time and a feeling of loss of control during these episodes. Two questions assessed purging, one assessed fasting, and three assessed excessive exercise. These questions were used to ascertain ED diagnoses based on DSM‐5 criteria, including threshold binge‐eating disorder (BED), subthreshold BED (S‐BED), threshold bulimia nervosa (BN), subthreshold BN (S‐BN), AN, and purging disorder (PD; Micali et al. 2015). These derived diagnoses are established variables in ALSPAC available for research access. For full algorithms used to determine diagnoses, please see Micali et al. (2015). We also collapsed timepoints and created an “any age” variable, such that if an individual had threshold, subthreshold, or any ED at any age it was coded present, and if not, it was coded absent.

### 2.4 Statistical Analyses

#### 2.4.1 Descriptive Analysis

Prevalence of pica at the 36‐, 54‐, 65‐, 77‐, and 115‐month assessments (Papini et al. 2024) and EDs at the 14‐ and 16‐year assessments (Micali et al. 2015) in the current sample have been previously reported. We calculated the frequency of EDs at 18 years of age based on definitions used in Micali et al. (2015) for EDs at 14 and 16 years of age. We examined the frequency of various DAWBA diagnoses and created cross‐tabulation tables for pica at 77‐ and 115‐months and DAWBA diagnoses (OCD, any anxiety disorder, any depressive disorder, any ADHD disorder, oppositional conduct disorder, pervasive developmental disorder) at 7.50 and 10.67 years, respectively. Supporting Information Tables 2 and 3 report the diagnostic frequency of DAWBA and ED observed in the current sample. We also created cross‐tabulation tables for ED diagnoses at the 14‐, 16‐, and 18‐year assessments and any pica and persistent pica.

After examining cross‐tabulations tables between pica and the separate DAWBA diagnoses, we observed very low cell counts not appropriate for further analyses. To protect participant confidentiality, and in alignment with the ALSPAC protocols, we have not reported these tables. Given low cell counts, we used the broader DAWBA diagnoses: any behavioral, any emotional, and any disorder at both 6.75‐ and 10.67 years of age calculated by ALSPAC. Any behavioral disorder consisted of any ADHD, oppositional defiant, conduct disorder, and hyperkinesis. Any emotional disorder consisted of OCD, any anxiety disorder (e.g., generalized, separation, social, phobia) and depressive disorders. Any disorder indicated the presence of any DAWBA‐diagnosed psychiatric disorder and is the combination of any emotional and any behavioral. We used these combined categories instead of individual disorder diagnoses in subsequent analyses.

We also observed low cell counts between pica_pers_ and ED outcomes. Consequently, we did not conduct logistic regressions using pica_pers_ as the predictor and only examined the prospective relationship between pica_any_ and EDs. Further, we collapsed ED diagnostic categories into threshold, threshold and subthreshold categories, and any ED. The threshold category includes those with threshold BED, BN, AN, or PD. Threshold and subthreshold includes those with threshold BED, BN, AN, or PD, and subthreshold BED and BN. Any ED includes those with threshold BED, BN, AN, or PD, and subthreshold BED and BN, and those with EDNOS. All categories are as defined by Micali et al. (2015).

Table 2 presents the cross‐tabulation of cross‐tabulation tables for behavioral, emotional, any DAWBA diagnosis, and EDs with pica across assessments that were used in analyses. Supporting Information Table 4 includes a full cross‐tabulation table between pica presence and ED diagnoses at the various ages.

#### 2.4.2 Aim 1

We conducted six logistic regression models to assess the relationship between pica and DAWBA diagnoses. We conducted three models for early childhood. Each used binary pica presence at 77 months for the predictor and a different DAWBA diagnosis (any behavioral disorder, any emotional disorder, and any disorder) at 7.50 years for the outcome. We conducted three models for mid‐childhood. Each mid‐childhood model used binary pica presence at 115 months for the predictor and a different DAWBA diagnosis (any behavioral disorder, any emotional disorder, and any disorder) at 10.67 years for the outcome. All models included sex as a covariate.

We conducted 12 linear regression models to assess the relationship between pica and SDQ scores. Six models were run for early childhood. Each early childhood model used binary pica presence at 77 months as the predictor and a different SDQ subscale score or the SDQ total score at 6.75 years for the outcome. Six models were run for mid childhood. Each mid childhood model used binary pica presence at 115 months as the predictor and a different SDQ subscale score or the SDQ total score at 115 months (about 9.78 years) for the outcome. All 12 models included sex as a covariate. We tested the assumptions of linear regression and found that the normality of residuals assumption was violated and that subscale scores exhibited skewness and kurtosis values outside of the acceptable range. To address these issues, we Box‐Cox transformed (using MASS R package; Venables and Ripley 2002) all the SDQ subscales at each time point and standardized to *z* scores. The *z* scores were then included as the outcome variable within the linear regression models. We calculated *sr* ^2^, the squared semipartial correlation, representing variance explained by that unique predictor in the model for each analysis.

#### 2.4.3 Aim 2

We conducted 12 logistic regressions to assess the relationship between pica (pica_any_) in childhood and EDs later in adolescence. There were four models for threshold ED, four models for threshold and subthreshold disorders, and four models for any ED. In each set, outcomes were assessed at 14, 16, and 18 years of age, and across any age. Each model used pica_any_ as the predictor. All analyses controlled for sex.

#### 2.4.4 Post Hoc‐Exploratory Analyses

After conducting the analyses for Aims 1 and 2, we conducted exploratory analyses to control for potential effects of child developmental level. At 42 months of age, children were assessed with the Denver Developmental Screening Test (Frankenburg and Dodds 1967). This screening tool helps identify children with developmental delays in personal‐social, fine motor and adaptive, language, and gross motor domains. We categorized the lowest decile as indicative of developmental delay (Papini et al. 2024). We then added this binary variable as a covariate in the Aim 1 SDQ Analyses.

All tests were conducted using *RStudio* (Version 2024.09.0 + 375; Posit 2024). We used listwise deletion, such that for each separate analysis we only included people who completed both measures. Therefore, each analysis varies in missingness. We conducted the Benjamini–Hochberg correction procedure, which controls for the False Discovery Rate (FDR; Benjamini and Hochberg 1995) to account for multiple testing. The correction was applied across all analyses conducted for Aims 1 and 2, treating all analyses as a single family of tests.

## 3 Results

### 3.1 Descriptives

The prevalence of pica was 2.33% at 38 months, 0.79% at 54 months, 0.65% at 65 months, 0.56% at 77 months, and 0.33% at 115 months. 3.10% of participants experienced pica any, and 0.65% had pica pers (Table 1).

**TABLE 1:** Prevalence of pica across development.

Timepoint	Count (*n*)	Frequency
38 months	230	2.33%
54 months	75	0.79%
65 months	56	0.64%
77 months	47	0.56%
115 months	25	0.33%
Persistant^a^	39	0.65%
Any^b^	190	3.10%

^a^ Persistant pica was coded as present if pica was endorsed at two or more time points.

^b^ Any pica was coded as present if pica was endorsed at any time point.

Supporting Information Tables 2 and 3 present the frequencies of DAWBA diagnoses at the 91‐ and 120‐month assessments, and EDs at 14‐, 16‐, and 18‐ years of age assessments, respectively.

#### 3.1.1 Psychopathology

##### 3.1.1.1 Aim 1

In early childhood, individuals with pica had greater odds of having any behavioral disorder (OR = 5.65, 95% CI = 2.23–12.43, *q* < 0.001) and greater odds of having any psychiatric disorder (OR = 7.30, 95% CI = 3.53–14.22, *q* < 0.001) than individuals without pica. In early childhood, individuals with pica also had greater odds of having any emotional disorder, although the CI was wide (OR = 4.07, 95% CI = 0.97–11.45, *q* = 0.039). A CI including zero typically indicates non‐significance. The discrepancy of the CI including zero but a *q* < 0.05 could be due to the low number of cases of individuals with both pica and any emotional disorder (Table 2), so this result should be interpreted cautiously.

**TABLE 2:** Frequency of co‐occurring conditions, stratified by, pica presence.

DAWBA diagnoses		Any behavioral		Any emotional		Any disorder		Any ED psychopathology any age^a^	
		Absent	Present	Absent	Present	Absent	Present	Absent	Present
77 months	Absent	6868	240	6999	132	6756	375		
	Present	34	**7**	38	**< 5** ^**b**^	29	**12**		
115 months	Absent	6330	210	6411	177	6217	317		
	Present	15	**6**	21	**< 5** ^**b**^	16	**6**		
Persistent pica^c^	Absent							1079	1794
	Present							5	**12**
Any pica^d^	Absent							1048	1750
	Present							36	**56**

*Note:* Bold values indicates to emphasis the cells indicating diagnositic overalp (ie.g., when people had both a DAWBA disorder and pica).

^a^ Those with threshold BED, BN, AN, or PD, and subthreshold BED and BN, and those with EDNOS and at‐risk for EDs, defined by Micali et al. (2015) at any age.

^b^ Cell counts < 5 may include zero.

^c^ Participants were coded to have any pica if they endorsed pica behaviors at any timepoint in childhood. Persistent pica represents pica behaviors endorsed at least two time points.

^d^ Any age is collapsed across ages 14, 16, or 18. For example, any age threshold is individuals who had threshold at 14, 16, and/or 18.

In mid‐childhood, individuals with pica had greater odds of having any behavioral disorder [OR = 10.66, 95% CI = 3.73, 26.86, *q* < 0.001] and greater odds of having any psychiatric disorder [OR = 5.75, 95% CI = 2.05, 14.15, *q* < 0.001] than individuals without pica. In mid‐childhood, individuals with pica also had greater odds of having any emotional disorder, although this difference was not statistically significant (OR = 1.71 95% CI = 0.10–8.27, *q* = 0.75) (Table 3 and Supporting Information Table 5). Reported *q* values represent *p* values after the FDR‐BH correction was conducted.

**TABLE 3:** Logistic regression results assessing the relationship between pica at 77 months of age and DAWBA^a^ diagnoses at 7.50 years of age or pica at 115 months of age and DAWBA diagnoses at 10.67 years of age using data from ALSPAC.^b^

		*B*	SE	*q* ^c^	OR	95% CI of OR
77 months	Any behavioral	1.73	0.43	**< 0.001**	5.65	[2.23, 12. 43]
	Any emotional	1.40	0.61	**0.039**	4.07	[0.97, 11.45]
	Any disorder	1.99	0.35	**< 0.001**	7.30	[3.53, 14.22]
115 months	Any behavioral	2.37	0.49	**< 0.001**	10.66	[3.73, 26.86]
	Any emotional	0.54	1.02	0.75	1.71	[0.10, 8.27]
	Any disorder	1.75	0.48	**< 0.001**	5.75	[2.05, 14.15]

*Note:* Pica presence was the predictor, DAWBA diagnosis the outcome, and sex was a covariate. Bold values indicates to highlight the tests that were statistically significant.

^a^ Development and well‐being assessment.

^b^ Avon longitudinal study of parents and children.

^c^ Reported *q* values reflect adjusted *p* values after Benjamini–Hochberg false discovery rate correction was conducted.

#### 3.1.2 Dimensional Psychopathology

##### 3.1.2.1 Aim 1

In early childhood, pica was significantly associated with all SDQ subscales and total SDQ score (Table 3). *R* ^2^ values were negligible for the emotional [*b* = 0.389, *sr* ^2^ = 0.00, *R* ^2^ = 0.004, *q* < 0.01], conduct [*b* = 0.88, *sr* ^2^ = 0.00, *R* ^2^ = 0.009, *q* < 0.01], and peer problems subscales [*b* = 0.74, *sr* ^2^ = 0.00, *R* ^2^ = 0.007, *q* < 0.01] and small for the prosocial [*b* = −0.80, *sr* ^2^ = 0.00, *R* ^2^ = 0.036, *q* < 0.01], hyperactivity [*b* = 0.83, *sr* ^2^ = 0.00, *R* ^2^ = 0.031, *q* < 0.01], and total difficulties subscales [*b* = 1.03, *sr* ^2^ = 0.01, *R* ^2^ = 0.017, *q* < 0.01] (Table 3; Cohen 1988). The presence of pica corresponded to a *z* score increase on the conduct problems (0.88), emotional problems (0.39), hyperactivity (0.83), peer problems (0.74), and total difficulties (1.03) subscales compared to no pica. Pica also corresponded to 0.80 z‐score points lower on the prosocial scale.

In mid‐childhood, pica was significantly associated with each SDQ subscale examined and total SDQ score. *R* ^2^ values were negligible for the emotional [*b* = 0.95, *sr* ^2^ = 0.00, *R* ^2^ = 0.010, *q* < 0.01], conduct [*b* = 0.76, *sr* ^2^ = 0.00, *R* ^2^ = 0.005, *q* < 0.01], and peer problems [*b* = 0.94, *sr* ^2^ = 0.00, *R* ^2^ = 0.004, *q* < 0.01] subscales, and small for the prosocial [*b* = −0.44, *sr* ^2^ = 0.00, *R* ^2^ = 0.038, *q* < 0.01] and hyperactivity subscales [*b* = 1.23, *sr* ^2^ = 0.00, *R* ^2^ = 0.033, *q* < 0.01] and total difficulties score [*b* = 1.45, *sr* ^2^ = 0.01, *R* ^2^ = 0.013, *q* < 0.01] (Table 3). Results indicate that pica was associated with a z‐score increase on the conduct problems (1.19 points higher), emotional problems (1.19), hyperactivity (1.23), peer problems (0.94), and total difficulties (1.45) subscales, indicating greater problems for children with pica. Pica was also associated with a *z* score 0.44 points lower on the prosocial scale, indicating worse prosocial skills for children with pica.

For both time periods, although the *z* score differences between groups were moderate to large both *R* ^2^ and *sr* ^2^ values associated with pica were negligible to small, indicating that pica explains a negligible to small amount of the variance in SDQ scores within the ALSPAC population (0.00 to 0.01; Table 4 and Supporting Information Table 6) and other variables contribute to SDQ score outcomes. Reported *q* values represent adjusted *p* values after the FDR‐BH correction was conducted.

**TABLE 4:** Linear regression results. For analyses labeled 77 months, pica presence at 77 months of age was the predictor, and various SDQ^a^ subscales at 6.75 years of age were the outcome. For analyses labeled 115 months, pica presence at 115 months of age was the predictor and various SDQ subscales at 115 months of age were the outcome. All analyses included sex as a covariate.

		77 months				115 months			
	Predictor	*b*	*β*	*sr* ^2^	Fit	*b*	*β*	*sr* ^2^	Fit
Conduct problems	Pica	0.88***	0.06	0.00		0.76***	0.04	0.00	
	Model fit				*R* ^2^ = 0.009** 95% CI [0.00, 0.01]				*R* ^2^ = 0.005** 95% CI [0.00, 0.01]
Emotional problems	Pica	0.39*	0.03	0.00		0.95***	0.05	0.00	
	Model fit				*R* ^2^ = 0.004** 95% CI [0.00, 0.01]				*R* ^2^ = 0.010** 95% CI [0.00, 0.01]
Hyperactivity	Pica	0.83***	0.06	0.00		1.23***	0.07	0.01	
	Model fit				*R* ^2^ = 0.031** 95% CI [0.02, 0.04]				*R* ^2^ = 0.033** 95% CI [0.03, 0.04]
Peer problems	Pica	0.74***	0.05	0.00		0.94***	0.05	0.00	
	Model fit				*R* ^2^ = 0.007** 95% CI [0.00, 0.01]				*R* ^2^ = 0.004** 95% CI [0.00, 0.01]
Prosocial	Pica	−0.80***	−0.06	0.00		−0.44*	−0.03	0.00	
	Model fit				*R* ^2^ = 0.036** 95% CI [0.03, 0.04]				*R* ^2^ = 0.038** 95% CI [0.03, 0.05]
Total difficulties	Pica	1.03***	0.07	0.01		1.45***	0.08	0.01	
	Model fit				*R* ^2^ = 0.017** 95% CI [0.01, 0.02]				*R* ^2^ = 0.013** 95% CI [0.03, 0.05]

*Note:* Reported statistical significance represents *q* values reflect adjusted *p* values after Benjamini–Hochberg false discovery rate correction was conducted for the predictors and represents *p* values for model fit.

^a^ Strengths and difficulties questionnaire.

*q < 0.05, **q < 0.01, ***q < 0.001; *p < 0.05, **p < 0.01, ***p < 0.001.

#### 3.1.3 Eating Disorders

##### 3.1.3.1 Aim 2

Logistic regression analysis revealed that the presence of pica did not significantly increase odds of having a threshold, subthreshold, or any ED diagnosis at 14‐, 16‐, and 18‐years of age (Table 5 and Supporting Informn Table 7). The reported *q* values in the table reflect adjusted *p* values after the FDR‐BH correction was conducted.

**TABLE 5:** Logistic regression results. All analyses had any pica^a^ as the predictor and eating disorder (ED) diagnoses categories were the outcomes.

		*B*	SE	*q* ^b^	OR [95% CI]
Threshold^c^	Age 14	−0.41	0.59	0.64	0.66 [0.16, 1.79]
	Age 16	−0.36	0.52	0.64	0.70 [0.21, 1.71]
	Age 18	−1.49	1.01	0.22	0.22 [0.01, 1.03]
	Any age	−0.49	0.38	0.30	0.61 [0.27, 1.23]
Threshold + subthreshold^d^	Age 14	−0.38	0.52	0.64	0.68 [0.21, 1.65]
	Age 16	−0.02	0.36	0.96	0.98 [0.45, 1.88]
	Age 18	−0.18	0.41	0.78	0.83 [0.34, 1.72]
	Any age	−0.02	0.38	0.96	0.98 [0.56, 1.66]
Any ED diagnosis^e^	Age 14	0.36	0.23	0.20	1.43 [0.89, 2.21]
	Age 16	−0.05	0.22	0.93	0.96 [0.62, 1.46]
	Age 18	0.04	0.35	0.96	1.04 [0.50, 1.97]
	Any age	−0.09	0.22	0.80	0.91 [0.59, 1.43]

^a^ Participants were coded to have any pica if they endorsed pica behaviors at any timepoint in childhood.

^b^ Reported *q* values reflect adjusted *p* values after Benjamini–Hochberg false discovery rate correction was conducted.

^c^ Threshold includes those with threshold BED, BN, AN, or PD, defined by Micali et al. (2015).

^d^ Threshold and subthreshold include those with threshold BED, BN, AN, or PD, and subthreshold BED and BN, defined by Micali et al. (2015).

^e^ Those with threshold BED, BN, AN, or PD, and subthreshold BED and BN, and those with EDNOS, defined by Micali et al. (2015).

### 3.2 Post Hoc Analyses

Given the large mean differences observed between those with and without pica on the SDQ subscales, but low variation explained in the linear regressions, we investigated whether having a developmental disability explained some of the difference found by including developmental disability as a covariate. See Supporting Information Table 8 for results.

Pica remained a significant predictor in the model after taking developmental delay into account. The *R* ^2^ values for each regression model did increase with the addition of developmental delay as a predictor. However, the *R* ^2^ values remained negligible to small, indicating the model explained a negligible to small percent (ranging from 1.2% to 5.4%) of the difference in means scores observed between those with and without pica. Further, the partial variance explained by the presence of a developmental disability was still negligible to small, with *sr* ^2^ ranging from 0.00 to 0.03 (see Supporting Information Table 8).

## 4 Discussion

The current study increases our understanding of psychiatric comorbidities of pica in childhood and later risk of eating disorder onset if pica was present in childhood. This is the first study to report concurrent comorbidities at more than one timepoint throughout development and the association between pica and later eating disorders. Furthermore, by using the ALSPAC dataset, a large‐scale population cohort, the current study provides improved estimates of psychiatric comorbidities of pica compared to potential biases potentially present in previous heterogeneous small‐scale studies. We found caregiver endorsement of pica behaviors in childhood (in early and mid‐childhood) increased odds of having a concurrent behavioral problem (ORs = 5.65 and 7.30, respectively) or any psychiatric problem (ORs = 10.66 and 5.75, respectively) at the diagnostic level (as measured by the DAWBA). We also found children with pica had greater conduct, emotional, prosocial, hyperactivity, and peer problems than children without pica. However, presence of pica behaviors did not significantly increase the odds of having an emotional disorder diagnosis. Furthermore, our findings suggest engaging in pica behaviors at any point throughout childhood was not associated with increased odds of developing an eating disorder, specifically BED, BN, AN, or PD in adolescence (14, 16, or 18 years of age).

Findings from the current study suggest pica may be associated with the risk of behavioral disorders rather than emotional disorders, indicating there might be common etiological mechanisms between pica and behavioral disorders. Pica did not statistically significantly increase the odds of an emotional disorder at the diagnostic level, but there was a small difference (*z* score mean difference of 0.39 in early childhood and 1.23 in mid‐childhood) in subthreshold dimensional psychopathology. The association between pica and emotional traits is significant but small (see aforementioned effect size). This indicates pica may be associated with emotional psychopathology at a trait level, but the effect size is not large enough for an individual to meet diagnostic criteria for an emotional disorder. It should be noted we observed low overlap between pica and emotional disorders in the current study, potentially precluding the ability to detect a significant relationship. The observed low overlap could be related to the relatively low prevalence of emotional disorders in childhood compared to adulthood (Center for Disease Control and Prevention 2023), and assessments at later stages of development may find significant associations between pica and emotional disorders.

In these analyses, we had to collapse categories, meaning we were unable to investigate specific mental health disorders. However, in the current study, pica corresponded to the greatest mean difference in hyperactivity out of the behavioral subscales measured by the SDQ. This analysis of dimensional traits provides a fine‐grain investigation in addition to the diagnostic‐level analyses. These findings support the theory that pica may be associated with greater ADHD and impulsivity, as children with pica in the current study reported greater hyperactivity—a key trait of ADHD—than children without pica (Hergüner and Hergüner 2010). The current study shows stronger support for a relationship between hyperactivity and pica rather than compulsivity and pica, as cross‐tabulation cell counts were low between pica and OCD and did not permit logistic regression analyses potentially indicating low comorbidity. Further, pica remained a significant predictor of psychopathology, even after we added developmental delay which, although significant, accounted for a small proportion of the variance. This study provides preliminary evidence that pica may be related to behavioral disorders and children with pica may struggle with more behavioral disorders.

The current results also suggest pica is associated with broad effects on psychopathology including peer relations, pro‐sociality, emotional, and behavioral health. Although pica and developmental delay explained only negligible to small amounts of the variance observed in behavioral problems between those with pica and those without pica, the moderate to large differences in mean scores imply individuals with pica are at increased risk of behavioral problems. This indicates that screening for behavioral problems among individuals with pica is more likely to be an effective screening strategy than screening for pica among children with behavioral problems.

The current results also suggest individuals with pica are not at increased odds of developing BED, BN, PD, or AN in mid to late adolescence. Unfortunately, the ALSPAC dataset did not contain information on avoidant/restrictive food intake disorder (ARFID) and rumination disorder diagnoses. Thus, we were unable to assess the association between pica and adolescent ARFID and rumination, which have previously shown the greatest overlap with pica (Hartmann et al. 2018; Murray et al. 2018). Results from other studies support the hypothesis that ARFID and pica share more overlap than with other eating disorders. Although our data suggest that there is not an increased risk of developing an eating disorder like AN, BN, PD, or BED, it will be important to replicate this finding in other population cohorts.

The study did have some limitations. First, although we had items to report pica behaviors, we acknowledge limitations in our measurement. As discussed in Papini et al. 2024, we could not derive a DSM‐5 pica diagnosis. Further, the self‐report items assessing pica limited the precision with which we could assess the frequency and severity of pica behaviors. Second, although the ALSPAC sample is large, we experienced small cell counts of symptom presentation and overlap, which limited statistical analysis options and statistical power. The small cell counts also limited the detail and nuance with which we could investigate psychiatric comorbidities. However, this was, in part, mitigated by the inclusion of dimensional psychopathology measures. We also could not assess the true concurrent existence of pica and psychopathology as the constructs were measured a few months apart in ALSPAC. Additionally, as noted in Papini et al. 2024, the ALSPAC cohort differs from the general population in the United Kingdom in that it is less diverse across socioeconomic status, race, and ethnicity than the general UK, potentially limiting the generalizability of the sample. It is also possible the prevalence and characteristics of pica have shifted since the start of the ALSPAC study, and children who currently present with pica exhibit different characteristics than children with pica born in the early 21st century.

To further our understanding of pica, future work would benefit from the creation of a questionnaire designed specifically to assess the diagnostic criteria of pica as described in the DSM‐5, potentially adapted from such measures as the Pica, ARFID, and Rumination Disorder Interview (PARDI; Bryant‐Waugh et al. 2022). Most questionnaires, as with the current study, assess for the consumption of non‐food substances but do not assess the other DSM‐5 diagnostic criteria of pica, including duration (i.e., at least 1 month) and inappropriateness given developmental age. Given the low prevalence of pica, future work would benefit from Likert‐type scales assessing frequency and severity of pica behaviors to increase statistical power and allow for a more precise understanding of pica behavior engagement and related characteristics. Further, future work should look at the longitudinal relationship between ARFID and pica and other eating disorders (e.g., BED and BN) across development. Also, as noted above, the ALSPAC sample is recruited from a limited geographic area in the United Kingdom. Although rates of psychopathology in the United Kingdom tend to match rates across other Western countries, future work should replicate investigations in different populations and cultures to assess the generalizability of results beyond the United Kingdom.

Overall, the current study provides preliminary evidence of the association of pica with behavioral disorder diagnoses and a broad range of childhood psychopathology. This furthers our knowledge of the clinical presentation of this understudied feeding and eating disorder, highlights the complex needs of individuals living with pica, and indicates the need for psychiatric screening when pica presents clinically. This initial work provides the basis for future work investigating psychiatric comorbidities with pica by providing insight into which disorders might be most common among those with pica and justification for future investigations into potential etiological overlap of pica and behavioral disorders. More longitudinal work is needed to understand the potential shared mechanisms between pica and behavioral disorders.

## Author Contributions

**Laura G. Rubino:** conceptualization, writing – original draft, writing – review and editing, formal analysis, methodology. **Cynthia M. Bulik:** conceptualization, resources, supervision. **Samuel J. R. A. Chawner:** conceptualization, funding acquisition, writing – original draft, writing – review and editing, supervision, resources, formal analysis, methodology. **Nadia Micali:** conceptualization, writing – review and editing, supervision, funding acquisition.

## Conflicts of Interest

Cynthia M. Bulik receives royalties from Pearson Education Inc. and served as a consultant for Orbimed. The other authors declare no conflicts of interest.

## Supporting information


**Data S1.** Tables.

## Data Availability

The data that support the findings of this study are available from ALSPAC. Restrictions apply to the availability of these data, which were used under license for this study. Data are available from the author(s) with the permission of ALSPAC.
