# Supplementary material for: Pica in Childhood: Concurrent and Sequential Psychiatric Comorbidity
Source: Int J Eat Disord. 2025 Jun 27;58(10):1936–45. doi: 10.1002/eat.24491 (PMC12398950; doi:10.1002/eat.24491)
Supplement: Supplementary file 1 — Data S1. Tables. [file EAT-58-1936-s001.docx]

**Supplemental Tables**

**Pica in childhood: concurrent and sequential psychiatric comorbidity**

Laura Rubino, Cynthia M. Bulik, Samuel JRA Chawner, Nadia Micali

| **Table 1.** Study Timeline illustrating the age in months or years participants (or their caregivers if a child) completed assessments for current study investigating pica in childhood and concurrent and sequential psychiatric comorbidity using data from ALSPAC^1^ | | | | |
| --- | --- | --- | --- | --- |
| Age | Pica | SDQ^2^ | DAWBA^3^ | Eating Disorder Psychopathology |
| 38 months | X |  |  |  |
| 54 months | X |  |  |  |
| 65 months | X |  |  |  |
| 77 months | X |  |  |  |
| 81 months |  | X |  |  |
| 91 months |  |  | X |  |
| 115 months | X | X |  |  |
| 128 months |  |  | X |  |
| 14 years |  |  |  | X |
| 16 years |  |  |  | X |
| 18 years |  |  |  | X |
| *^1^Avon Longitudinal Study of Parents and Children*  *^2^Strengths and Difficulties Questionnaire*  *^3^Development and Well - Being Assessment* | | | | |

| **Table 2.** DAWBA^1^ diagnosis frequency among children in ALSPAC^2^ | | | | |
| --- | --- | --- | --- | --- |
|  | 7 years old | | 10 years old | |
|  | Count | Proportion | Count | Proportion |
| OCD^3^ | 9 | 0.11% | <5 | 0.05% |
| Conduct disorder | 47 | 0.58% | 44 | 0.61% |
| Oppositional defiant disorder | 167 | 2.07% | 246 | 3.37% |
| Any anxiety | 251 | 3.10% | 152 | 2.07% |
| Any depressive | 39 | 0.48% | 70 | 0.97% |
| Any ADHD^4^ | 172 | 2.13% | 118 | 1.61% |
| Any emotional | 173 | 2.14% | 202 | 2.74% |
| Any behavioral | 296 | 3.65% | 253 | 3.47% |
| Any disorder | 463 | 5.85% | 437 | 6.02% |
| *^1^DAWBA = Development and Well-Being Assessment (Goodman et al., 2000)*  *^2^Avon Longitudinal Study of Parents and Children*  *^3^OCD = obsessive-compulsive disorder*  *^4^ADHD = attention deficit/hyperactivity disorder* | | | | |

| **Table 3.** Prevalence of eating disorders among adolescents in ALSPAC^1^ | | | | | |  |  |  |  |
| --- | --- | --- | --- | --- | --- | --- | --- | --- | --- |
|  |  | Age 14 | | Age 16 | | Age 18 | | Any Age*^5^* | |
|  |  | Count (*n*) | Percent | Count (*n*) | Percent | Count | Percent | Count | Percent |
| Threshold^2^ | BED | 28 | 0.48 | 55 | 1.14 | 53 | 1.67 | 121 | 4.80 |
|  | BN | 16 | 0.27 | 38 | 0.79 | 24 | 0.75 | 197 | 7.67 |
|  | PD | 24 | 0.41 | 75 | 1.55 | 54 | 1.70 | 137 | 5.40 |
|  | AN | 147 | 2.51 | 88 | 1.82 | 42 | 1.32 | 241 | 9.35 |
| Subthreshold^3^ | BED | <5* | NA | 23 | 0.48 | 140 | 4.40 | 163 | 6.49 |
|  | BN | 77 | 1.31 | 160 | 3.31 | 21 | 0.66 | 117 | 4.66 |
| Any ED^4^ |  | 1595 | 27.24 | 1929 | 39.90 | 1248 | 39.22 | 3277 | 76.89 |
| **cell counts <5 may include zero*  *^1^Avon Longitudinal Study of Parents and Children*  *^2^Threshold includes those with threshold BED, BN, AN, or PD, defined by Micali et al., 2015*  *^3^ Sub-threshold includes those with sub-threshold BE or BN defined by Micali et al., 2015*  *^4^Includes those with threshold BED, BN, AN, or PD, and sub-threshold BED and BN, and those with EDNOS and at-risk for EDs, defined by Micali et al., 2015*  *^5^Any age is collapsed across ages 14, 16, or 18. For example, any age threshold is individuals who had threshold at 14, 16, and/or 18.* | | | | | | | | | |

| **Table 4.** Frequency of co-occurring conditions stratified by pica presence among adolescents in ALSPAC^1^ | | | | | | | | |
| --- | --- | --- | --- | --- | --- | --- | --- | --- |
| Eating Disorders | | | Threshold^2^ | | Threshold and Sub-threshold^3^ | | Any ED psychopathology^4^ | |
|  |  |  | Absent | Present | Absent | Present | Absent | Present |
| Age 14 | Persistent Pica^5^ | Absent | 4053 | 162 | 4005 | 210 | 2490 | 725 |
|  |  | Present | 24 | **<5*** | 24 | **<5*** | 21 | **<5*** |
|  | Any Pica^5^ | Absent | 3964 | 159 | 3917 | 206 | 3421 | 702 |
|  |  | Present | 113 | **<5*** | 112 | **<5*** | 90 | **26** |
| Age 16 | Persistent Pica | Absent | 3408 | 184 | 3291 | 301 | 2299 | 1293 |
|  |  | Present | 18 | **<5*** | 15 | **<5*** | 7 | **11** |
|  | Any Pica | Absent | 3325 | 180 | 3210 | 295 | 2239 | 1266 |
|  |  | Present | 101 | **<5*** | 96 | **<5*** | 67 | **38** |
| Age 18 | Persistent Pica | Absent | 2300 | 124 | 2177 | 247 | 2133 | 291 |
|  |  | Present | 16 | **<5*** | 15 | **<5*** | 15 | **<5*** |
|  | Any Pica | Absent | 2238 | 123 | 2120 | 241 | 2079 | 282 |
|  |  | Present | 78 | **<5*** | 72 | **>5*** | 69 | **10** |
| Any age^6^ | Persistent Pica | Absent | 1773 | 403 | 1637 | 627 | 1079 | 1794 |
|  |  | Present | 11 | **<5*** | 9 | **<5*** | 5 | **12** |
|  | Any Pica | Absent | 1726 | 395 | 1595 | 612 | 1048 | 1750 |
|  |  | Present | 58 | **8** | 51 | **19** | 36 | **56** |
| **Cell counts <5 may include zero*  *^1^Avon Longitudinal Study of Parents and Children*  *^2^ Threshold includes those with threshold BED, BN, AN, or PD, defined by Micali et al., 2015*  *^3^Threshold and sub-threshold includes those with threshold BED, BN, AN, or PD, and sub-threshold BED and BN, defined by Micali et al., 2015*  *^4^Includes those with threshold BED, BN, AN, or PD, and sub-threshold BED and BN, and those with EDNOS and at-risk for EDs, defined by Micali et al., 2015*  *^5^Participants were coded to have any pica if they endorsed pica behaviors at any timepoint in childhood. Persistent pica represents pica behaviors endorsed at least 2 time points*  *^6^Any age is collapsed across ages 14, 16, or 18. For example, any age threshold is individuals who had threshold at 14, 16, and/or 18.* | | | | | | | | |

| **Table 5**. Logistic Regression Results assessing the relationship between pica at 77 months of age and DAWBA*^1^* diagnoses at 7.50 years of age or pica at 115 months of age and DAWBA diagnoses at 10.67 years of age using data from ALSPAC*^2^.* Pica presence was the predictor, DAWBA diagnosis the outcome, and sex was a covariate. | | | | | | | | | | |
| --- | --- | --- | --- | --- | --- | --- | --- | --- | --- | --- |
|  |  | B | SE | *q** | OR | 95% CI of OR | χ² | Wald’s test | AIC | df |
| 77 months | Any behavioral | 1.73 | 0.43 | **<0.001** | 5.65 | [2.23, 12. 43] | 91.4 | 0.00 | 2046.6 | 7135 |
|  | Any emotional | 1.40 | 0.61 | **0.039** | 4.07 | [0.97, 11.45] | 10.3 | 0.006 | 1336.9 | 7158 |
|  | Any disorder | 1.99 | 0.35 | **<0.001** | 7.30 | [3.53, 14.22] | 79.6 | <0.001 | 2936 | 7158 |
| 115 months | Any behavioral | 2.37 | 0.49 | **< 0.001** | 10.66 | [3.73, 26.86] | 54.6 | <0.001 | 1848.8 | 6550 |
|  | Any emotional | 0.54 | 1.02 | 0.75 | 1.71 | [0.10, 8.27] | 0.31 | 0.86 | 1643.2 | 6599 |
|  | Any disorder | 1.75 | 0.48 | **<0.001** | 5.75 | [2.05, 14.15] | 32.3 | <0.001 | 2862.1 | 6599 |
| **Reported q values reflect adjusted p values after Benjamini-Hochberg False Discovery Rate correction was conducted*  *^1^Development and Well - Being Assessment*  *^2^Avon Longitudinal Study of Parents and Children* | | | | | | | | | |  |

| **Table 6.** Linear Regression Results. For analyses labeled 77 months, pica presence at 77 months of age was the predictor and various SDQ^1^ subscales at 6.75 years of age were the outcome. For analyses labeled 115 months, pica presence at 115 months of age was the predictor and various SDQ subscales at 115 months of age were the outcome. All analyses included sex as a covariate. | | | | | | | | | | | |
| --- | --- | --- | --- | --- | --- | --- | --- | --- | --- | --- | --- |
|  |  | 77 months | | | | | 115 months | | | | |
|  | Predictor | *b* | *beta* | *sr^2^* | *r* | Fit | *b* | *beta* | *sr^2^* | *r* | Fit |
| Conduct problems | Pica | 0.88*** | 0.06 | 0.00 | 0.07 |  | 0.76*** | 0.04 | 0.00 | 0.05 |  |
|  | sex | -0.13** | -0.07 | 0.00 | -0.07 |  | -0.10** | -0.05 | 0.00 | -0.05 |  |
|  | Model fit |  |  |  |  | *R^2^*  = .009** |  |  |  |  | *R^2^*  = .005** |
|  |  |  |  |  |  | 95% CI[.00,.01] |  |  |  |  | 95% CI[.00,.01] |
| Emotional problems | Pica | 0.39* | 0.03 | 0.00 | 0.03 |  | 0.95*** | 0.05 | 0.00 | 0.05 |  |
|  | sex | 0.11** | 0.05 | 0.00 | 0.05 |  | 0.17** | 0.08 | 0.01 | 0.08 |  |
|  | Model fit |  |  |  |  | *R^2^*  = .004** |  |  |  |  | *R^2^*  = .010** |
|  |  |  |  |  |  | 95% CI[.00,.01] |  |  |  |  | 95% CI[.00,.01] |
| Hyperactivity | Pica | 0.83*** | 0.06 | 0.00 | 0.06 |  | 1.23*** | 0.07 | 0.01 | 0.08 |  |
|  | sex | -0.33** | -0.16 | 0.03 | -0.17 |  | -0.33** | -0.17 | 0.03 | -0.17 |  |
|  | Model fit |  |  |  |  | *R^2^*  = .031** |  |  |  |  | *R^2^*  = .033** |
|  |  |  |  |  |  | 95% CI[.02,.04] |  |  |  |  | 95% CI[.03,.04] |
| Peer problems | Pica | 0.74*** | 0.05 | 0.00 | 0.05 |  | 0.94*** | 0.05 | 0.00 | 0.06 |  |
|  | sex | -0.12** | -0.06 | 0.00 | -0.06 |  | -0.07** | -0.04 | 0.00 | -0.04 |  |
|  | Model fit |  |  |  |  | *R^2^*  = .007** |  |  |  |  | *R^2^*  = .004** |
|  |  |  |  |  |  | 95% CI[.00,.01] |  |  |  |  | 95% CI[.00,.01] |
| Prosocial | Pica | -0.80*** | -0.06 | 0.00 | -0.06 |  | -0.44* | -0.03 | 0.00 | -0.03 |  |
|  | sex | 0.36** | 0.18 | 0.03 | 0.18 |  | 0.39** | 0.19 | 0.04 | 0.19 |  |
|  | Model fit |  |  |  |  | *R^2^*  = .036** |  |  |  |  | *R^2^*  = .038** |
|  |  |  |  |  |  | 95% CI[.03,.04] |  |  |  |  | 95% CI[.03,.05] |
| Total difficulties | Pica | 1.03*** | 0.07 | 0.01 | 0.08 |  | 1.45*** | 0.08 | 0.01 | 0.09 |  |
|  | sex | -0.22** | -0.11 | 0.01 | -0.11 |  | -0.15** | -0.08 | 0.01 | -0.08 |  |
|  | Model fit |  |  |  |  | *R^2^*  = .017** |  |  |  |  | *R^2^*  = .013** |
|  |  |  |  |  |  | 95% CI[.01,.02] |  |  |  |  | 95% CI[.01,.02] |
| **q<0.05, **q<0.01, *** q<0.001; *p<0.05, **p<0.01, ***p<0.001*  **Reported statistical significance represents q values reflect adjusted p values after Benjamini-Hochberg False Discovery Rate correction was conducted for the predictors and p values for model fit ^1^Strengths and Difficulties Questionnaire* | | | | | | | | | | | |

| **Table 7**. Logistic Regression Results, All analyses had Any Pica^1^ as the predictor and Eating Disorder Diagnoses (ED) categories were the outcomes. | | | | | | | |
| --- | --- | --- | --- | --- | --- | --- | --- |
|  |  | B | SE | q | OR | AIC | Df model |
| Threshold^2^ | Age 14 | -0.41 | 0.59 | .64 | 0.66 [0.16, 1.79] | 1359.2 | 4236 |
|  | Age 16 | -0.36 | 0.52 | .64 | 0.70 [0.21, 1.71] | 1392.6 | 3607 |
|  | Age 18 | -1.49 | 1.01 | .22 | 0.22 [0.01, 1.03] | 947.88 | 2437 |
|  | Any age | -0.49 | 0.38 | .30 | 0.61 [0.27, 1.23] | 2043.3 | 2184 |
| Threshold + sub-threshold*^3^* | Age 14 | -0.38 | 0.52 | .64 | 0.68 [0.21, 1.65] | 1648.5 | 4236 |
|  | Age 16 | -0.02 | 0.36 | .96 | 0.98 [0.45, 1.88] | 1981.7 | 3607 |
|  | Age 18 | -0.18 | 0.41 | .78 | 0.83 [0.34, 1.72] | 1556.6 | 2437 |
|  | Any age | -0.02 | 0.38 | .96 | 0.98 [0.56, 1.66] | 2615.9 | 2274 |
| Any ED diagnosis*^4^* | Age 14 | 0.36 | 0.23 | .20 | 1.43 [0.89, 2.21] | 3801.2 | 4236 |
|  | Age 16 | -0.05 | 0.22 | .93 | 0.96 [0.62, 1.46] | 434.9 | 3607 |
|  | Age 18 | 0.04 | 0.35 | .96 | 1.04 [0.50, 1.97] | 1723 | 2437 |
|  | Any age | -0.09 | 0.22 | .80 | 0.91 [0.59, 1.43] | 3668.4 | 2887 |
| *^1^Participants were coded to have any pica if they endorsed pica behaviors at any timepoint in childhood ^2^ Threshold includes those with threshold BED, BN, AN, or PD, defined by Micali et al., 2015*  *^3^Threshold and sub-threshold includes those with threshold BED, BN, AN, or PD, and sub-threshold BED and BN, defined by Micali et al., 2015*  *^4^Includes those with threshold BED, BN, AN, or PD, and sub-threshold BED and BN, and those with EDNOS, defined by Micali et al., 2015* | | | | | | | |

| **Table 8.** Post Hoc Linear Regression Results. For analyses labeled 77 months, pica presence at 77 months of age was the predictor and various SDQ^1^ subscales at 6.75 years of age were the outcome. For analyses labeled 115 months, pica presence at 115 months of age was the predictor and various SDQ subscales at 115 months of age were the outcome. All analyses included sex and likely presence of a developmental disability (DD) as covariates | | | | | | | | | | | |
| --- | --- | --- | --- | --- | --- | --- | --- | --- | --- | --- | --- |
|  |  | 77 months | | | | | 115 months | | | | |
|  | Predictor | *b* | *beta* | *sr^2^* | *r* | Fit | *b* | *beta* | *sr^2^* | *r* | Fit |
| Conduct problems | Pica | 1.61** | 0.08 | 0.01 | 0.09 |  | 1.79** | 0.07 | 0.00 | 0.07 |  |
|  | DD | 0.32** | 0.07 | 0.00 | 0.08 |  | 0.28** | 0.06 | 0.00 | 0.07 |  |
|  | sex | -0.14** | -0.05 | 0.00 | -0.06 |  | -0.14** | -0.05 | 0.00 | -0.06 |  |
|  | Model fit |  |  |  |  | *R^2^*  = .015** |  |  |  |  | *R^2^*  = .012** |
|  |  |  |  |  |  | 95% CI[.01,.02] |  |  |  |  | 95% CI[.01,.02] |
| Emotional problems | Pica | 0.63* | 0.03 | 0.00 | 0.03 |  | 1.49** | 0.05 | 0.00 | 0.05 |  |
|  | DD | 0.50** | 0.09 | 0.01 | 0.08 |  | 0.60** | 0.11 | 0.01 | 0.09 |  |
|  | sex | 0.21** | 0.06 | 0.00 | 0.05 |  | 0.34** | 0.10 | 0.01 | 0.08 |  |
|  | Model fit |  |  |  |  | *R^2^*  = .011** |  |  |  |  | *R^2^*  = .020** |
|  |  |  |  |  |  | 95% CI[.01,.02] |  |  |  |  | 95% CI[.01,.03] |
| Hyperactivity | Pica | 2.24** | 0.07 | 0.00 | 0.08 |  | 3.45** | 0.08 | 0.01 | 0.09 |  |
|  | DD | 1.15** | 0.15 | 0.02 | 0.17 |  | 1.07** | 0.14 | 0.02 | 0.17 |  |
|  | sex | -0.66** | -0.14 | 0.02 | -0.16 |  | -0.67** | -0.15 | 0.02 | -0.18 |  |
|  | Model fit |  |  |  |  | *R^2^*  = .054** |  |  |  |  | *R^2^*  = .059** |
|  |  |  |  |  |  | 95% CI[.04,.06] |  |  |  |  | 95% CI[.05,.07] |
| Peer problems | Pica | 1.63** | 0.09 | 0.01 | 0.09 |  | 2.17** | 0.08 | 0.01 | 0.09 |  |
|  | DD | 0.69** | 0.14 | 0.02 | 0.16 |  | 0.87** | 0.17 | 0.03 | 0.18 |  |
|  | sex | -0.14** | -0.05 | 0.00 | -0.07 |  | -0.07 | -0.02 | 0.00 | -0.05 |  |
|  | Model fit |  |  |  |  | *R^2^*  = .034** |  |  |  |  | *R^2^*  = .039** |
|  |  |  |  |  |  | 95% CI[.03,.04] |  |  |  |  | 95% CI[.03,.05] |
| Prosocial | Pica | -1.70** | -0.07 | 0.01 | -0.08 |  | -1.50** | -0.05 | 0.00 | -0.06 |  |
|  | DD | -0.79** | -0.13 | 0.02 | -0.16 |  | -0.63** | -0.11 | 0.01 | -0.14 |  |
|  | sex | 0.53** | 0.15 | 0.02 | 0.17 |  | 0.57** | 0.17 | 0.03 | 0.19 |  |
|  | Model fit |  |  |  |  | *R^2^*  = .054** |  |  |  |  | *R^2^*  = .052** |
|  |  |  |  |  |  | 95% CI[.04,.06] |  |  |  |  | 95% CI[.04,.06] |
| Total difficulties | Pica | 5.81** | 0.09 | 0.01 | 0.10 |  | 8.87** | 0.10 | 0.01 | 0.11 |  |
|  | DD | 2.63** | 0.16 | 0.03 | 0.18 |  | 2.80** | 0.17 | 0.03 | 0.19 |  |
|  | sex | -0.73** | -0.08 | v.01 | -0.10 |  | -0.54** | -0.06 | 0.00 | -0.09 |  |
|  | Model fit |  |  |  |  | *R^2^*  = .045** |  |  |  |  | *R^2^*  = .047** |
|  |  |  |  |  |  | 95% CI[.04,.06] |  |  |  |  | 95% CI[.04,.06] |
| **p<0.05, **p<0.01, *** p<0.001*  *^1^Strengths and Difficulties Questionnaire* | | | | | | | | | | | |
